# L-thyroxine modifies nephrotoxicity by regulating the apoptotic pathway: The possible role of CD38/ADP-ribosyl cyclase-mediated calcium mobilization

**DOI:** 10.1371/journal.pone.0184157

**Published:** 2017-09-11

**Authors:** Tarek El-Hamoly, Dina M. El-Sharawy, Marwa S. El Refaye, Sahar S. Abd El-Rahman

**Affiliations:** 1 Drug Radiation Research Department, National Center for Radiation Research and Technology, Atomic Energy Authority, Cairo, Egypt; 2 Cyclotron Project, Center of Nuclear Researches, Atomic Energy Authority, Cairo, Egypt; 3 Department of Pathology, Faculty of Veterinary Medicine, Cairo University, Giza, Egypt; Faculty of Medicine & Health Science, UNITED ARAB EMIRATES

## Abstract

Thyroid hormones are well-established as a key regulator of many cellular metabolic pathways developed in various pathogeneses. Here, we dedicated the current work to investigate the role of thyroid hormone analogue (L-thyroxine, L-TH) in regulating the renal cytotoxicity using *in vivo* and *in vitro* models. Swiss mice were exposed to gamma radiation (IRR, 6Gy) or treated with cisplatin (CIS, 15 mg/kg, *i*.*p*.*)* for induction of nephrotoxicity. Remarkably, pretreatment with L-TH (1μg/kg) ameliorated the elevated kidney function biomarkers, oxidative stress and protected the renal tissue from the subsequent cellular damage. Likewise, L-TH inhibited the apoptotic cascade by down-regulating the extreme consumption of the cellular energy (ATP), the expression of caspase-3 and Bax, and the stimulation of cyclic ADP ribose (cADPR)/calcium mobilization. Moreover, incubation with L-TH (120nM/4h) significantly blocked the cytotoxicity of CIS on Vero cells and the depletion of NAD+ content as well as modified the ADP-ribose cyclase (CD38) enzymatic activity. High doses of L-TH (up to30 nM/4h) inversely increased the radiosensitivity of Vero cells towards IRR (up to 6Gy). On the other hand, L-TH did not interfere CIS-induced cytotoxicity of colorectal adenocarcinoma (Caco-2) cell line. In conclusion, pretreatment with L-TH could be a promising protective approach to the renal cellular damage induced during either *CIS* or IRR therapy by regulating the unbalanced oxidative status, the expression of pro-apoptotic biomarkers via modulation of cADPR mediated-calcium mobilization.

## Introduction

Clinical strategies based on narrow therapeutic range still an outstanding issue provoking the successful treatment of malignant diseases. Cell cycle non-specific drugs (e.g. cisplatin and other alkylating agents) that mainly act by inhibiting all stages of tumor cells proliferation, except G_0_-resting phase, follow first-order kinetics in dose-response curve. Owing to the use of CIS in high doses to achieve its effect, the manifestation of nephrotoxicity side effect is much abundant in malignant individuals, which limits its clinical use. During repeated administration regimen, CIS generates reactive oxygen species (ROS), impairs the antioxidant defense system, stimulates excessive poly (ADP-ribose) polymerization (PARylation) and triggers the caspase-dependent apoptosis in normal renal cells [1–3]. Moreover, positively charged CIS metabolites typically accumulate within the mitochondria [4]; the action that contributes in mitochondrial perturbation by preventing respiratory chain complexes (I–IV) [5]. Using CIS analogues (e.g. carboplatin and oxaliplatin), however, may be one possible approach with less marked renal toxicity. On the other hand, kidney is a dose-limiting organ that much affected by dose and source of ionizing radiation in the course of abdominal malignancies radiotherapy. Potential protective strategies including combined adjuvants therapy (e.g. Amifostine) have been introduced in the treatment of solid tumors to avoid such prominent nephropathy. They are exerting their effects by neutralizing the platinum-reactive metabolites, scavenging free radicals, accelerating DNA repair, inhibiting apoptosis, and regulating other gene expressions in normal renal cells [6, 7]. Despite of these innovative approaches in tumor therapy, the efficient treatment for neoplastic diseases without adverse reactions still represents an unmet clinical need. Nicotinamide adenine dinucleotide (NAD+) is an essential cofactor that facilitates the progression of cellular pathways. It is a common substrate for three main consuming enzymes namely; poly (ADP-ribosyl) polymerases (PARPs), sirtuins (SIRTs) and cADP-ribose synthases (CD38 and CD157). Importantly, these NADases express different stoichiometric affinity for NAD+ at different compartments in living cells and their modified stimulation interfere with many abnormalities [8]. Thyroid hormones (THs) considered as important controller of several physiological pathways including transcriptional factors activation, cellular proliferation and maturation and mitochondrial biogenesis via mitophagy [9–11]. By virtue of their basal metabolic rate stimulation, THs have been implicated in diverse biological models ranging from different ischemia/reperfusion (I/R) [12], diabetic nephropathy [13], hepatotoxicity [14], and other inflammatory responses [15]. Based on TH-related reactions (oxidative stress response and apoptosis regulation) in different pathological disorders, we set out to find out the possible signaling pathways through which the prohormone (L-thyroxine, L-TH) could influence CIS- or IRR-mediated nephrotoxicity.

## Materials and methods

### Chemicals

Cisplatin (CIS, cis-diammine platinum (II) dichloride, Mylan, Canonsburg, PA, USA).

Levothyroxine sodium (L-TH, Sigma-aldrich, St. Louis, MO, USA), it was freshly prepared by dissolving in small amount of alkaline solution and finally diluted by sterile 0.9% NaCl solution.

### Animals and nephrotoxicity induction

All procedures were conducted in accordance with the protocol approved by the Institutional Animal Care and Use Committee at Cairo University (IACUC, CUIIS916), Egypt. Forty-eight male Swiss mice (weighing 20-25g) were obtained from Animal House belongs to Nile Pharmaceutical Company, Cairo, Egypt. The animals were housed under controlled conditions with 12h light/dark cycle, allowed free access to standard food pellets and distilled water. Animals were left one week for acclimatization on laboratory conditions prior to the onset of the experiment. Nephrotoxicity was induced in two groups either by acute whole body IRR at dose level of 6 Gy using the gamma cell irradiator (CM-20, Co^60^, Russia), Cyclotron project, Atomic Energy Authority, Egypt or mice were intraperitoneally (*i*.*p*) administered 15mg/kg of cisplatin [16].

### Experimental protocol

Mice were randomly allocated into six experimental groups (n = 8): G1; control negative group, G2; sham operated group, mice were *i*.*p* administrated L-TH (1μg/kg), G3; mice were administrated single dose of CIS as mentioned before, G4; mice were exposed to IRR as previously mentioned. While mice of G5 and G6 were administered L-TH (1μg/kg) four or one hours before induction of nephrotoxicity by CIS administration or IRR exposure respectively. Animals were sacrificed 72h following CIS administration or 24h post irradiation exposure. Blood samples were collected under gentle diethyl-ether anesthesia prior to scarification and kidney tissue samples were collected for further biochemical and histopathological examinations.

### Kidney functions evaluation

Serum creatinine, uric acid and urea levels were evaluated according to manufacturer's instruction of the colorimetric kit (Bio-diagnostic Co., Cairo, Egypt).

### Oxidative stress evaluation

Hydrogen peroxide (H_2_O_2_) elimination was measured in kidney tissue homogenates as an indicator of oxidative stress using colorimetric kit (Bio-diagnostic Co., Cairo, Egypt). The absorbance was detected at 510nm by a UVD-2950 spectrophotometer. Catalase activity was also assayed according to the method of **Luck** [17]. The changes in absorbance were recorded at 240 nm/min/5min and the activity was calculated as unit/mg protein.

### DNA fragmentation assay

DNA was isolated and purified according to QIAamp manufacturer's protocol (QIAGEN, Hilden, Germany). The extracted DNA were separated by electrophoresis on 2% agarose gel containing 1 μg/mL ethidium bromide and visualized by UV transillumination.

### Determination of cell integrity (serum LDH activity)

Serum lactate dehydrogenase was measured according to the manufacturer’s instructions of commercial kit (Salucea, Haansberg, Netherland), whereas LDH activity was estimated as mean absorbance change/min/dl at 340 nm.

### Cell culture and viability assay (MTT)

Cryopreserved Vero (Green African monkey kidney) cells were obtained from the Cell Bank of VACSERA (Cairo, Egypt). All chemicals used were obtained from (Sigma-Aldrich, St. Louis, MO, USA). Cells were cultured in a humidified atmosphere (5%CO_2_, 37°C) with RPMI-1640 Medium supplemented with 2% fetal bovine serum (FBS) and 50μg/ mL gentamycin. Cells were passaged at 80–90% confluency after trypsinization with pre-warmed trypsin-EDTA solution. Four experiments were carried out on Vero cells. The first two were conducted for determination of the IC_50_ of both CIS and L-TH, at which Vero cells in one experiment were incubated with different concentrations of CIS for 24 hours, while in the second experiment, they were pretreated with different doses of L-TH for 30min before incubation with CIS (60μM/24h).

In the third experiment, cells were pretreated with 120nM of L-TH for different time periods (30 min, 1h, 2h, 3h and 4h), and then incubated with 60 μM CIS to determine the time-dependent effect of L-TH exposure. In the last experiment, cells were pretreated with different doses of L-TH before exposure to gamma radiation at dose levels of 2 or 6 Gy. At the end of all experiments, 1 X 10^5^ cells were either subjected to cytotoxicity test or used for further chromatographic analysis.

To investigate whether the pretreatment with L-TH affecting the CIS-induced colorectal carcinoma cells (Caco-2) cytotoxicity, the Human colorectal adenocarcinoma (Caco-2) cells were used. Cells were purchased from the Cell Bank of VACSERA (Cairo, Egypt) and maintained in high glucose DMEM supplemented with 10% FBS and 1% penicillin-streptomycin at 37°C in a humidified atmosphere of 5% CO_2_. They were pretreated (4h) with either L-TH or PARP inhibitor (5-AIQ) before incubation with CIS. Cell viability (MTT) were determined according to the quantitative colorimetric assay with 3-(4,5 dimethylthiazol-2-yl)-2,5-diphenyltetrazoli-umbro-mide (MTT) as previously described by **Lakatos et al.** [18]. The absorbance of living cells was measured at 560 nm.

### HPLC analysis of nucleotides and cytosolic calcium estimation

Mitochondria and cytosols were separated according to the fractionation protocol of **Dimauro et al.,** [19]. Free cytosolic calcium levels were evaluated using inductive coupled plasma-optical emission spectroscopy (ICP-OES, Ultima Expert, HORIBA, France) and expressed as μg/mg protein. On mitochondrial fraction of kidney tissue samples from different groups, the ATP content was acid/base extracted from mitochondrial fractions and then quantified using high performance liquid chromatography (HPLC) according to the method of [20]. The HPLC system with UV detector (Sykam, Germany) was set according to the condition of separation: C_18_ column (Hypersil, USA), flow rate: 0.5ml/min, temperature: 25°C, injector volume: 20 μl, run time: 10 min, mobile phase: 0.2M KH_2_PO_4_: acetonitrile: methanol (9.6:0.3:0.1). The peaks were separated at λ = 254 nm. The concentration of ATP was calculated using a calibration curve and expressed as μg/mg protein. NAD^+^ were extracted and determined in Vero cells, pretreated with either L-TH or PARP inhibitor (5-AIQ) before CIS incubation, by a reverse-phase HPLC method [21]. NAD^+^ peaks were observed at 261 nm and expressed as μM/mg protein content of cultured cells. Cyclic (ADP) ribose activity was evaluated in either kidney tissues or Vero cells. Extraction and chromatographic analysis of the produced cADPR (an indicator of ADP-ribosyl cyclase) were carried out as mentioned by [22]. https://www.protocols.io/private/e54bd98cd8056e2f515ee981051ed956

### Histopathological and immunohistochemical studies

Kidney specimens from different experimental groups were fixed in 10% buffered neutral formalin. Afterward, formalin fixed specimens were routinely dehydrated in graded series of ethanol, cleared in xylol for conventional paraffin embedding technique. Paraffin sections of about 4–5 μm were stained with H&E [23]. Four point numerical scoring system has been used to express the degree of severity of the observed histopathological lesions (where 0 indicates no change and 1, 2, and 3 indicate mild, moderate, and severe changes respectively), while the grading was determined by percentage according to **Arsad et al.,** [24] as follows: changes less than 30% (<30%) indicating mild changes, changes less than 30–50% (<30–50%) indicating moderate changes and changes more than 50% (>50%) indicating severe changes.

Immunohistochemical studies were carried out for detection of Caspase-3, Bax and CD38 expressions on paraffin sections of kidney using avidin-biotin peroxidase (DAB, Sigma Chemical Co.) according to the method described by **Hsu et al.,** [25]. Briefly, tissue sections were incubated with a monoclonal antibody for caspase-3, Bax and CD38 (Dako Corp, Carpenteria, CA) and reagents required for the avidin-biotin peroxidase (Vactastain ABC peroxidase kit, Vector Laboratories) method for the detection of the antigen–antibody complex. Each marker expression was localized by the chromagen 3,3-diaminobenzidine tetrahydrochloride (DAB, Sigma Chemical Co.).

### Protein determination

Protein contents were estimated for all samples using BCA protein assay kit (Pierce, Rockford, IL, USA).

### Statistical analysis

All values are presented as means ± SEM. Statistical analysis of experimental data was performed with one-way analysis of variance (ANOVA), followed by Tukey's Multiple Comparison Test. Results were considered statistically significant when at least *P< 0*.*05*.

## Results

### L-TH improves the altered kidney functions and oxidative status in mouse model of nephrotoxicity

**Fig 1:**
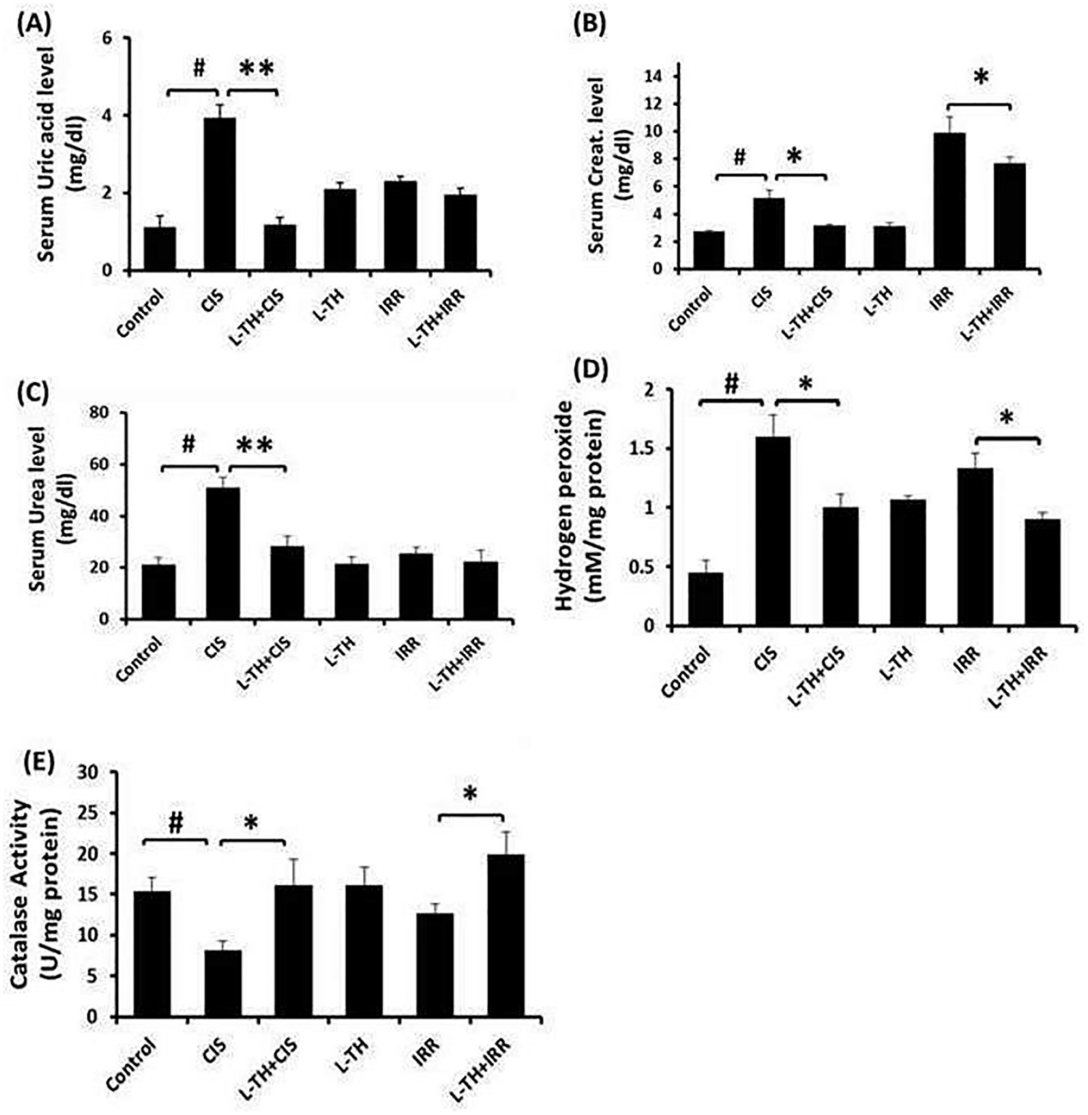
Effect of L-TH on serum uric acid (A), creatinine (B) and urea nitrogen (C) levels as well as biochemical analysis of oxidative status; H_2_O_2_ content (D) and catalase activity (E) in kidney tissues of all experimental groups. Acute renal injury was induced in male Swiss mice by single *i*.*p*. injection of cisplatin (15mg/kg) or gamma irradiation (6 Gy). Animals were pretreated with L-TH (1μg/kg) four hours before induction of nephrotoxicity. L-TH- and vehicle-treated mice were served as normal controls. Values are expressed as mean ± SEM (n = 6–8). ^*#*^*P*< 0.05: significantly different versus vehicle treated control; *P< 0.05, **P< 0.01: significantly different versus untreated control. CIS, cisplatin; H_2_O_2_, hydrogen peroxide; IRR, gamma irradiated; L-TH, L-thyroxine.

Cisplatin administration resulted in marked renal dysfunction after 72h, as was evident by significant **(***P<* 0.05) high serum levels of uric acid, creatinine, and urea nitrogen compared with normal control. Likewise, exposure to gamma radiation resulted in significant **(***P<* 0.05) rise in creatinine and uric acid levels 24h post irradiation exposure without any effect on urea nitrogen. On contrary, the pretreatment with L-TH significantly **(***P<* 0.05) decreased the elevated levels of renal function biomarkers in both models (Fig 1A, 1B and 1C). Both administration of CIS and exposure to gamma radiation caused three-fold significant **(***P<* 0.05) increase in renal H2O2 content and catalase activity compared to control group. While, L-TH pre-administration resulted in significant antioxidant effect as evidenced by significant decrease in hydrogen peroxide levels as well as significant increase in catalase activity in CIS and IRR models, respectively (Fig 1D and 1E).

### Protective effect of L-TH pretreatment against CIS- and IRR-induced mitochondrial ATP depletion, cellular integrity damage and DNA fragmentation

**Fig 2:**
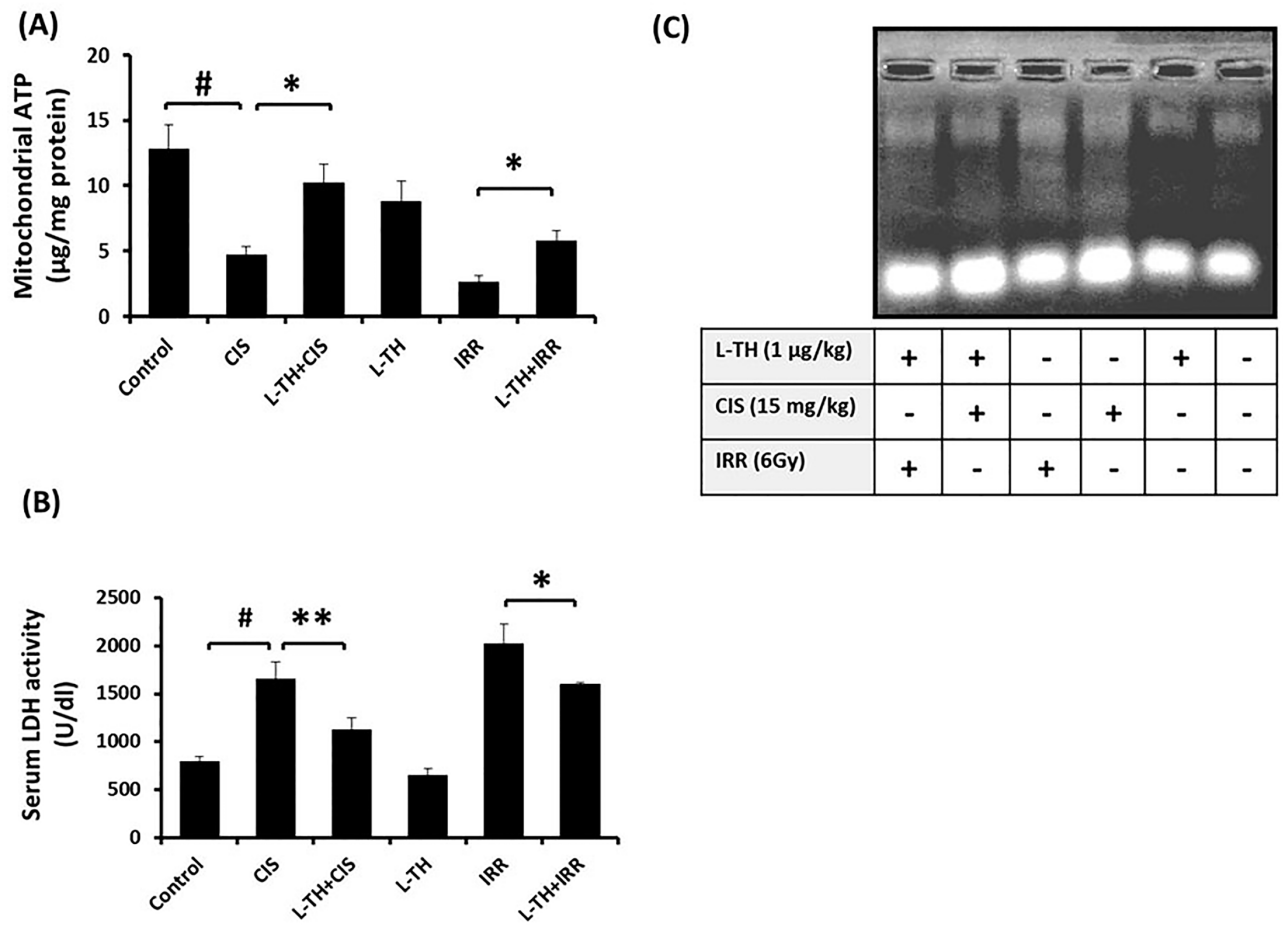
**(A)** HPLC separation of ATP contents was performed in isolated mitochondrial fractions of renal tissue via isocratic method. Cell death biomarkers**;** serum LDH activity (**B**) and DNA fragmentation **(C)** were determined in both models of nephrotoxicity. Error pars represent SEM for six independent experiments. ^*#*^ *P<* 0.05: significantly different versus vehicle treated control; *P< 0.05, **P< 0.01: significantly different versus untreated control.

Excessive ATP consumption and ROS production is a checkpoint of apoptotic pathway. As shown in Fig 2A, two-fold decrease in mitochondrial ATP content was observed after mice had been exposed to whole body IRR (6Gy) or treated with CIS. The pretreatment with L-TH was likely conserved the mitochondrial ATP content in both models. The increased plasma membrane permeability as indicated by serum LDH is a sign of cell death as well. It was observed that, administration of CIS or IRR exposure caused LDH release into the circulation. While pretreatment with L-TH slightly decreased the release of LDH in IRR mice, and completely abolished its release and plasma membrane disruption in CIS treated ones (Fig 2B). Similarly, a discharge of nuclear DNA fragments and appearance of DNA smear was demonstrated in kidney samples of CIS-administered and gamma radiation-exposed mice, the phenomenon that inhibited upon pretreatment with L-TH (Fig 2C).

### Histopathological results

**Fig 3:**
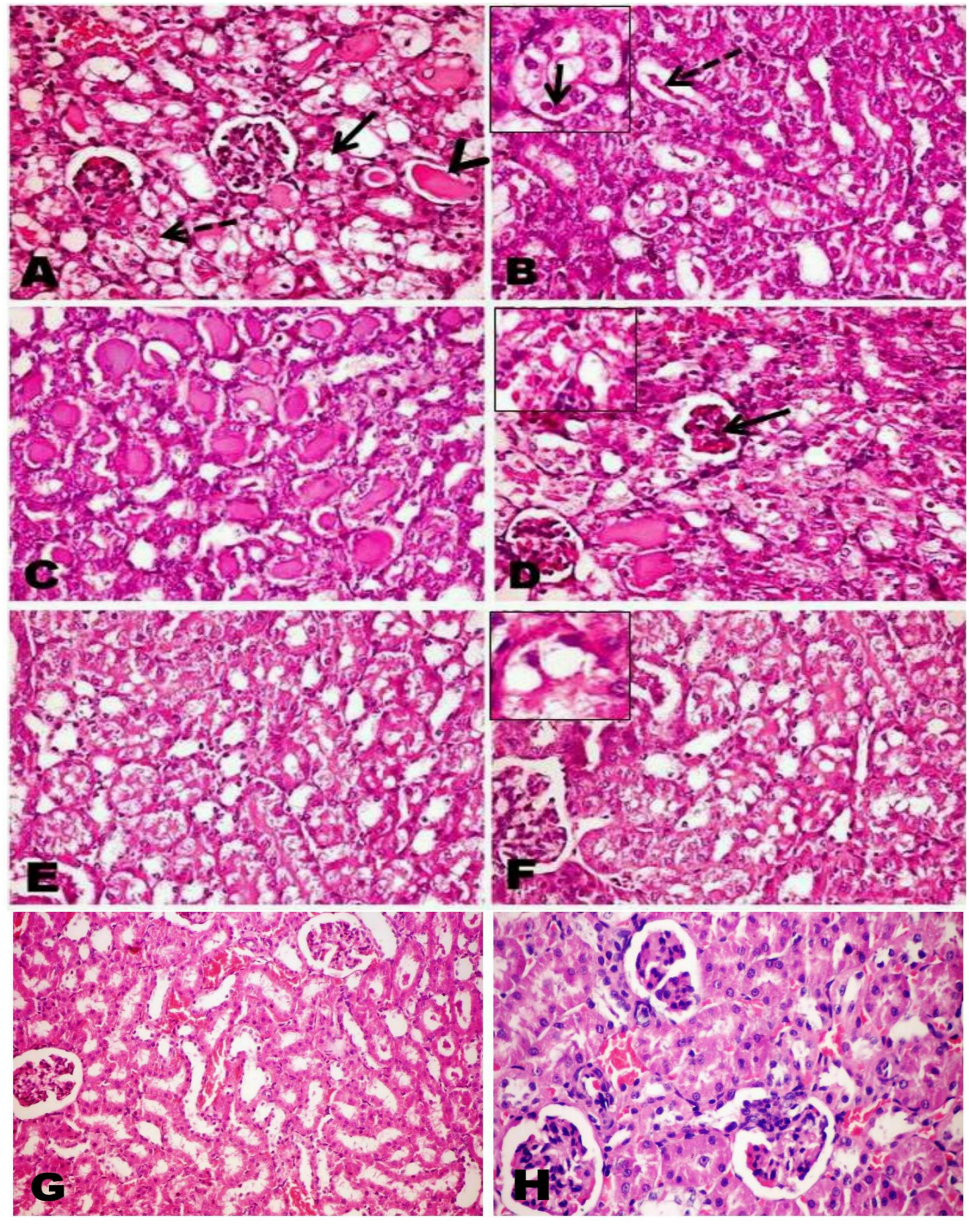
(A-D) Kidneys of CIS (15mg/kg) administrated mouse showing (A) vacuolar degeneration (arrow) and necrosis of the renal tubular epithelial linings, hyaline droplets (dashed arrow) and hyaline cast (arrow head). (B) Many apoptotic cells and bodies (arrow) with presence of granular cast (dashed arrow) in the lumen of renal tubules. (C) Wide spread hyaline cast (H) in the lumen of the medullary tubules. (D) Glomerular congestion, hyperplasia of the podocytes and shrinkage of the glomerular tuft (arrow), notice the wide spread of hyaline droplets (upper left) in many tubules. (E-F) Kidney of IRR (6 Gy) mouse showing marked swelling, granular and vacuolar (upper left) degeneration of the renal tubular epithelial linings with many necrotic cells and granular cast (arrow) in the lumen of some tubules. (G) Kidney of CIS and L-TH pretreated mouse showing mild degeneration of the renal tubular epithelium with scattered necrotic cells and small granular cast in the lumen of few tubules (arrow). (H) Kidney of IRR and L-TH pretreated mouse showing good restoration of renal tissue with only mild tubular epithelial degenerative changes. (H&E, X400).

Microscopic examination of different kidney sections of both control and L-TH- treated mice revealed normal histological structure. While, kidney sections of both nephrotoxicity models showed marked tissue alterations. Regarding renal tissue of CIS administrated mice, the renal tubules especially the proximal ones showed widespread vacuolar degeneration and necrosis of the epithelial linings (Fig 3A). The necrotic cells appeared either with pyknotic nuclei or without any nuclear structure or desquamated with the presence of hyaline droplets and granular cast in the tubular lumens. Many apoptotic cells and bodies were noticed (Fig 3B). Hyaline casts were conspicuously observed in the lumen of the medullary tubules (Fig 3C). Congestion and variable sizes areas of intertubular hemorrhages were also seen. The glomeruli showed vascular congestion, hyperplasia of the podocytes as well as shrinkage of the glomerular tufts of large number of glomeruli (Fig 3D). Moderate thickening of the parietal layer of the Bowman’s’ capsule was noticed in some animals. On the other hand, exposure to gamma radiation resulted in deleterious effects on renal histology; marked swelling, granular and vacuolar degeneration of the renal tubular epithelial linings (Fig 3E and 3F) with many necrotic cells were all observed. Some desquamated cells were accumulated in the lumen of the renal tubules forming granular cast. Marked conservation of the renal tissue against the unwholesome effects of CIS as well as IRR exposure was observed upon pretreatment with L-TH. There was mild to moderate degenerative changes of the renal tubular epithelium with scattered necrotic cells. Rare small granular casts were noticed in the lumen of some tubules. The glomerular changes caused by CIS were greatly declined with near to absence of the apoptotic bodies (Fig 3G). Moreover, only mild degenerative and necrotic changes were observed among the renal tubular epithelium in kidney of irradiated rats which pretreated with L-TH (Fig 3H). The severity of the observed histopathological lesions in different groups were summarized and scored in Table 1. Values showed that; the pretreatment with L-TH revealed marked decrease in the intensity or even disappearance of some of the histopathological lesions induced by both insults of nephrotoxicity.

**Table 1:** Summary of the histopathological findings in various groups with its scoring.

	The observed lesion	Control Group		L-TH Group		Cisplatin Group		Cisplatin and L-TH Group		Gamma irradiation Group		Gamma irradiation and L-TH Group	
		N	Nu	N	Nu	N	Nu	N	Nu	N	Nu	N	Nu
**Tubular changes**	**Vacuolar degeneration of the tubular epithelium**	**0**	**0**	**1**	**0.14±0.14**	**7**	**3**^a^^b^	**7**	**1.4±0.2** ^c^	**7**	**3**^a^^b^	**7**	**1.4±0.2**^d^
	**Tubular epithelial necrosis**	**0**	**0**	**1**	**0.14±0.14**	**7**	**3**^a^^b^	**7**	**1.2±0.18**^c^	**7**	**3**^a^^b^	**7**	**1.2±0.18**^d^
	**Apoptotic cells and bodies**	**0**	**0**	**0**	**0**	**7**	**2.5±0.2**^a^^b^	**4**	**0.7±0.18**^c^	**3**	**0.4±0.2**^a^^b^	**0**	**0**
	**Hyaline droplets**	**0**	**0**	**0**	**0**	**7**	**2.7±0.18**^a^^b^	**2**	**0.28±0.18**^c^	**0**	**0**	**0**	**0**
	**Hyaline cast**	**0**	**0**	**0**	**0**	**7**	**3**^a^^b^	**2**	**0.28±0.18**^c^	**0**	**0**	**0**	**0**
**Glomerular changes**	**Congestion and hyperplasia of podocytes**	**0**	**0**	**2**	**0.2±0.18**	**7**	**3**^a^^b^	**4**	**0.57±0.2**^c^	**7**	**3**^a^^b^	**5**	**0.71±0.2**^d^
	**Glomerular shrinkage**	**0**	**0**	**0**	**0**	**7**	**1.8±0.2**^a^^b^	**3**	**0.4±0.2**^c^	**0**	**0**	**0**	**0**
	**Thickening of the parietal layer**	**0**	**0**	**0**	**0**	**4**	**0.8±0.3**^a^^b^	**2**	**0.2±0.18**	**0**	**0**	**0**	**0**
**Interstitial changes**	**Congestion of the intertubular blood capillaries**	**0**	**0**	**1**	**0.14±0.14**	**7**	**1.7±0.18**^a^^b^	**7**	**1**^c^	**7**	**1.8±0.2**	**6**	**1**^d^
	**Intertubular pockets oh hemorrhage**	**0**	**0**	**0**	**0**	**7**	**1.4±0.2**^a^^b^	**2**	**0.2±0.18**^c^	**4**	**1.4±0.3**^a^^b^	**2**	**0.2±0.18**^d^

N: Number of animals showed the lesion, Nu: Numerical score.

Data are expressed as mean ± SE (n = 7).

^**a**:^ Significantly different from corresponding control group at *P≤ 0*.*05*

^**b**:^ Significantly different from corresponding T3 administrated group at *P≤ 0*.*05*

^**c**:^ Significantly different from corresponding cisplatin administrated group at *P≤ 0*.*05*

^**d**:^ Significantly different from corresponding gamma irradiation exposed group at *P≤ 0*.*05*

### Pre-administration of L-TH protects against CIS- or IRR-induced apoptosis

**Fig 4:**
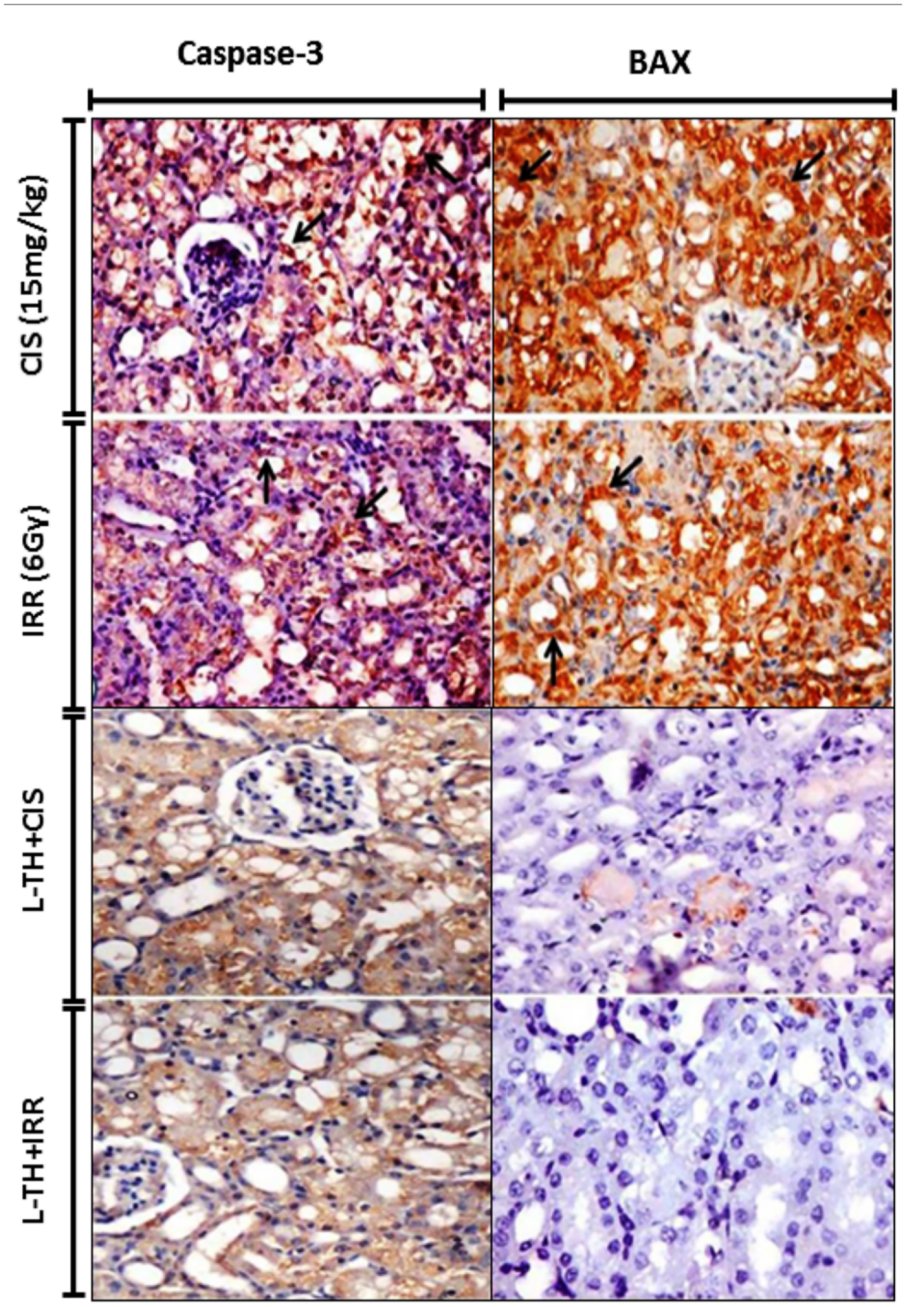
L-TH protects against CIS and IRR-induced apoptosis. Immunohistochemistry for caspase-3 and Bax showing positive expression among the renal tubular epithelium in both models of nephrotoxicity. Very mild to negative expressions of caspase-3 and Bax among the tubular epithelial linings in kidneys of pre-administered L-TH/cisplatin or L-TH/gamma irradiated mice (X 400).

Immunohistochemistry staining of different kidney sections revealed marked positive expression of both caspase-3 and Bax among the renal tubular epithelial linings of cisplatin administrated as well as gamma irradiated mice. An obvious decrease of both apoptotic markers expressions was noticed in kidney sections from L-TH pretreated mice (Fig 4).

### ADP-ribosyl cyclase activity in nephrotoxicity

**Fig 5:**
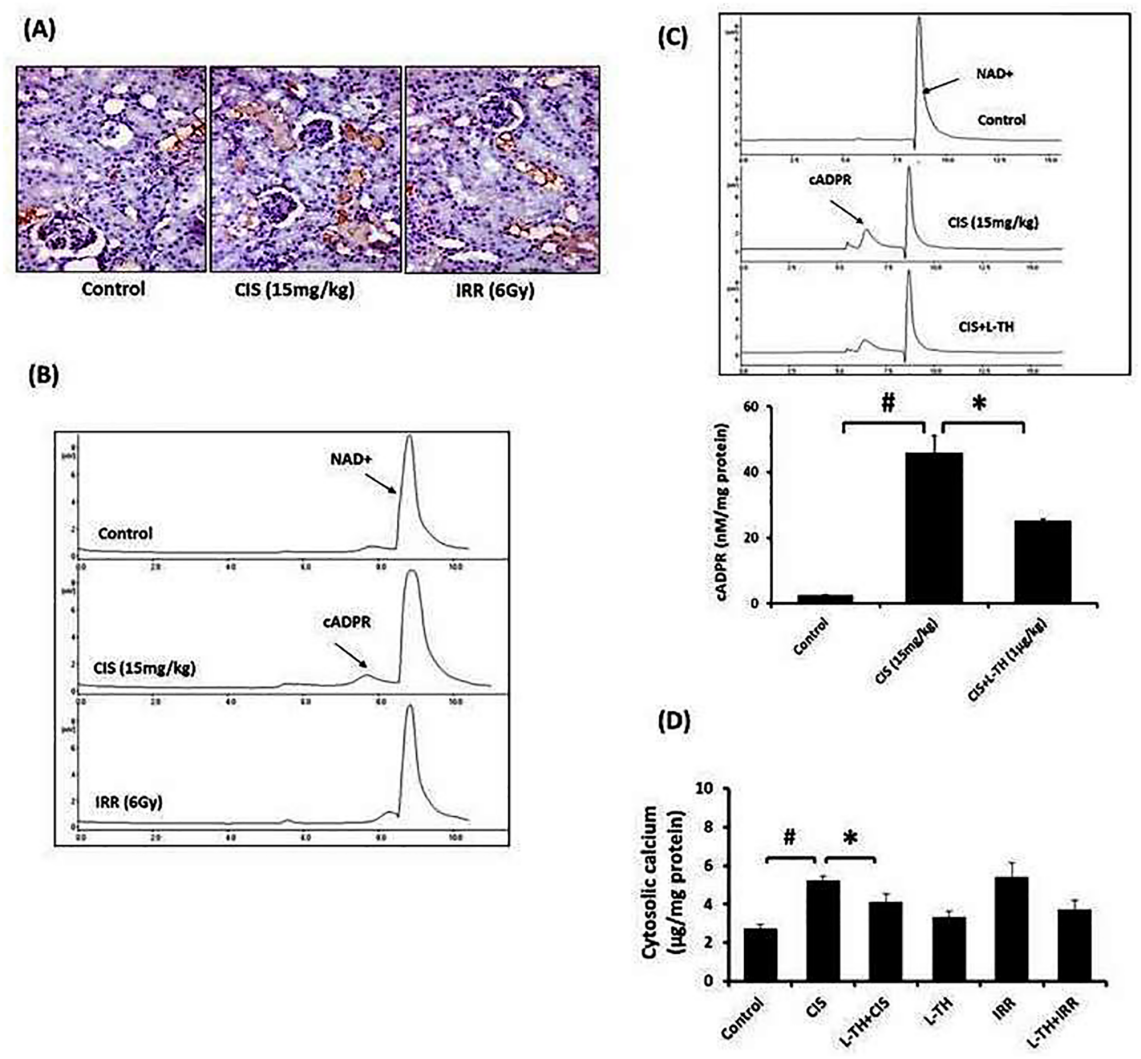
L-TH regulates CD38 activity through formation of cADPR and calcium mobilization. **(A)** Immunohistochemistry demonstrated that; CD38 expression was predominant in tubular epithelium of renal tissue and this was unchanged by either CIS administration or gamma radiation exposure. **(B and C)** Chromatogram of cADPR and NAD^+^ indicated high CD38 (ADP-ribosyl cyclase) activity. Extracted cADPR and NAD^+^ contents were reverse phase HPLC evaluated according to the linear gradient separation conditions. (**D)** Calcium contents were quantified in isolated cytosolic fraction of kidney samples using ICP-OES. Error pars represent SEM for six independent experiments. ^*#*^*P* <0.05: significantly different versus vehicle treated control; *P<0.05: significantly different versus untreated control.

To determine the location of CD38 in the kidney, Immunohistochemistry (IHC) was performed on control, CIS- and IRR- nephrotoxic kidneys. High expression of CD38 was observed in the renal tubular epithelium of all of the experimental groups (Fig 5A). However, the activity of CD38 was intensely increased in CIS-treated mice, indicated by high peak of separated cADPR (Fig 5B). Pre-treatment with L-TH significantly decreased such elevated cADPR; the effect that may inhibit the free calcium liberation into cytosol (Fig 5C and 5D).

### Effect of L-TH on CIS or IRR-induced Vero cells cytotoxicity

**Fig 6:**
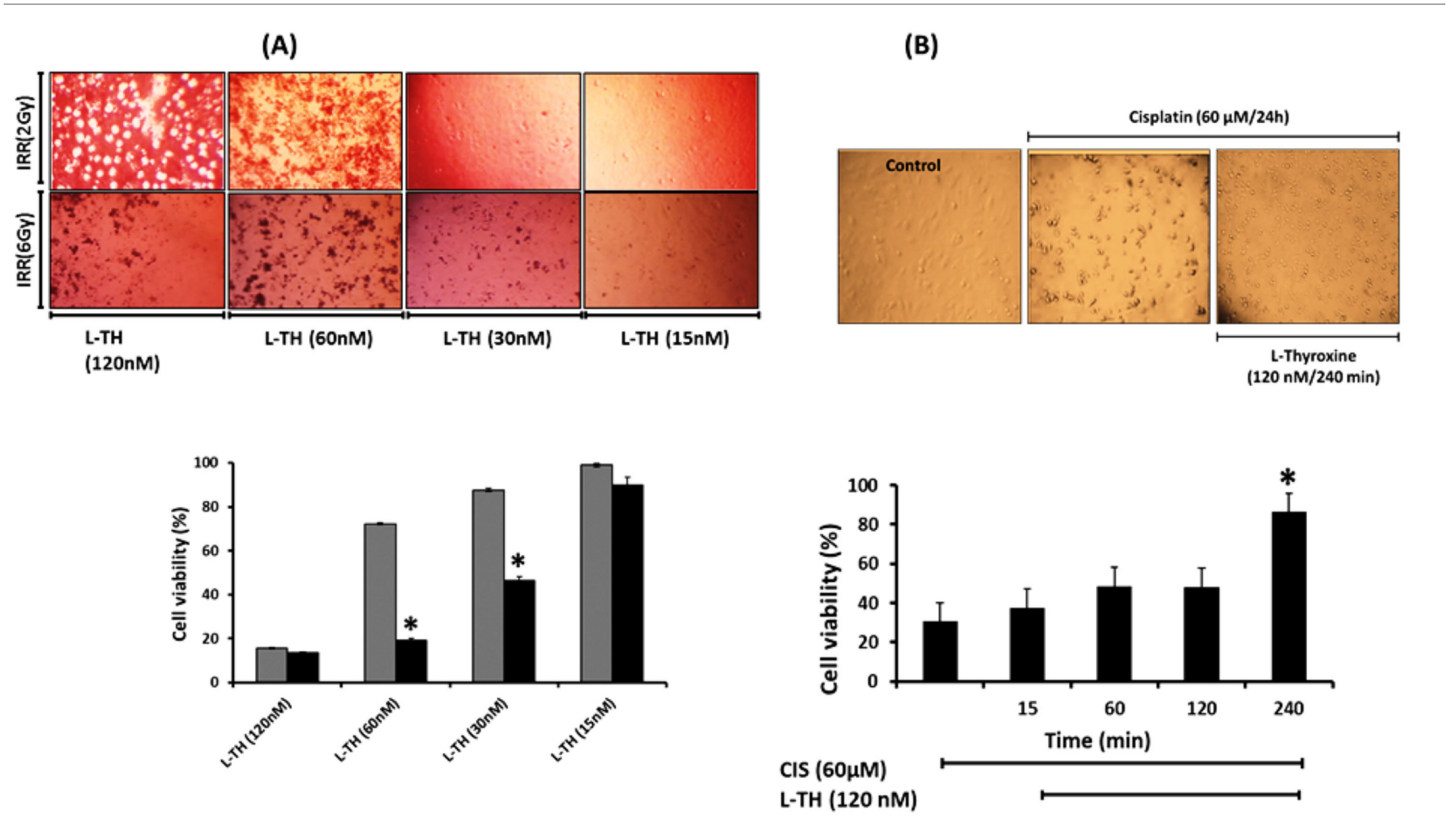
Pretreatment with L-TH antagonizes cisplatin-induced cellular death and modified the radiosensitivity. **(A)** Vero cells were pretreated with L-TH (120 nM) for different exposure time before incubation with cisplatin (60μM/24h). L-TH protected the cells from cisplatin induced dose-dependent loss of viability and L-TH protected the cells from cisplatin-induced cell death in time-dependent effect. **(B)** The cells were pretreated with different doses of L-TH two hours before they were IRR with either 2 or 6 Gy dose levels. Cell viability was determined using MTT assay after 24h incubation. Data are expressed as mean (% of control) ± SEM of three independent experiments. (*) indicates significant difference versus CIS-treated or 2Gy-irradiated cells (*P< 0.05).

Vero cells could tolerate gamma irradiation up to the dose level of 6 Gy. Interestingly, the pretreatment with high doses of L-TH showed a radio-sensitizing effect toward IRR, indicated by loss of Vero cells viability irradiated with either 2Gy or 6Gy dose levels (Fig 6A). However, changing the doses of L-TH alone or with CIS-treated cells did not have any cytotoxic effect on Vero cells (S1 Fig). Moreover, the pretreatment with L-TH could survive cells in time-dependent manner from CIS-mediated suppression of viability (Fig 6B).

### L-TH modulates the ADP-ribosyl cyclase and NAD^+^

**Fig 7:**
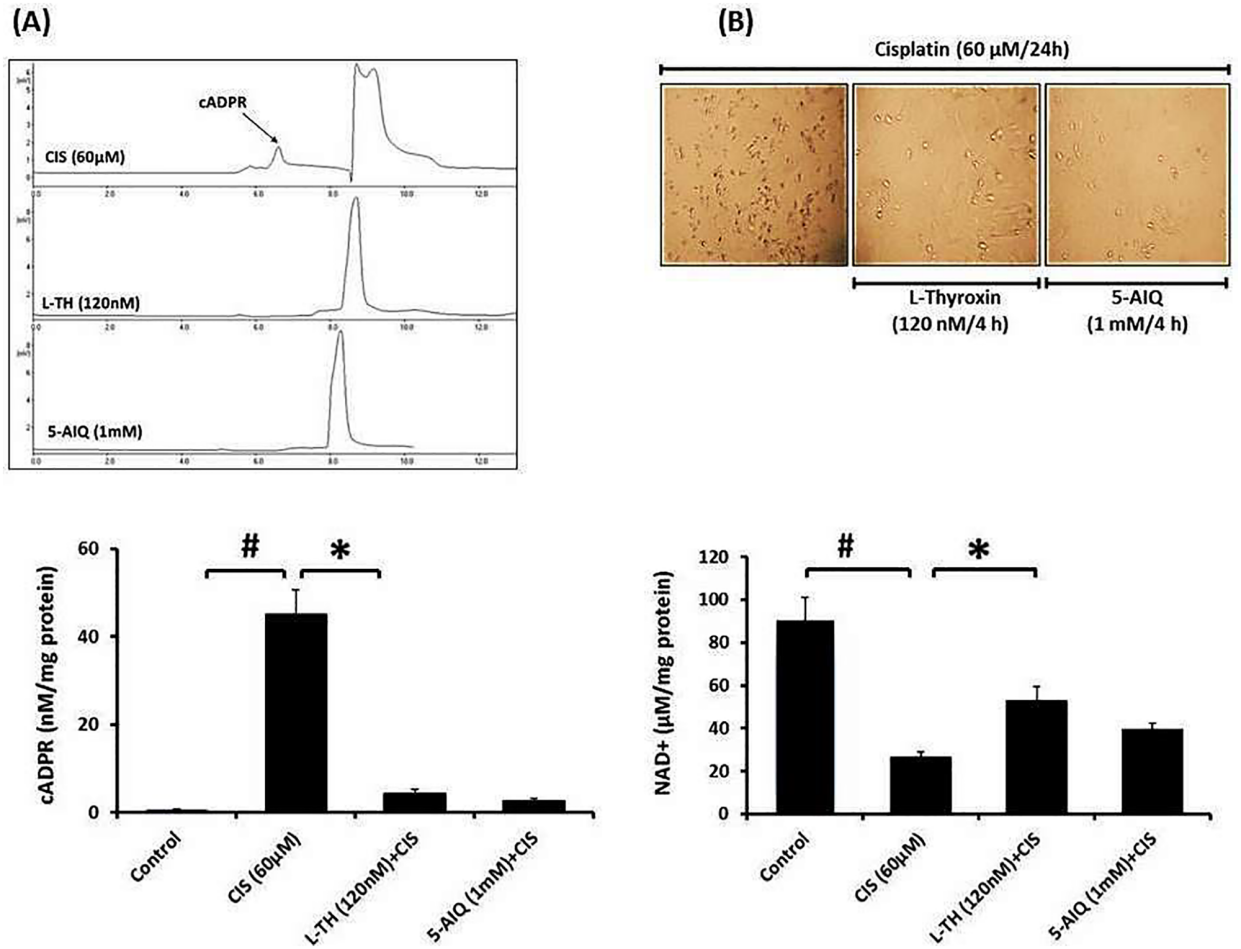
**(A)** Vero cells were treated with either L-TH/CIS or 5-AIQ/CIS. Extracted NAD+ was HPLC separated according to the linear gradient protocol; flow rate: 1 ml/min, temperature: 25°C, mobile phase: 0.05 M phosphate buffer: methanol (gradient ratio for 30 min), λ = 261nm. **(B)** Cells (1X10^5^/sample) were incubated for 60 min, at 37°C with or without 0.1 mM β-NAD^+^. cADPR was subsequently extracted and analyzed by HPLC system according to linear gradient separation conditions; mobile phase: 0.1M KH_2_PO_4_ containing 5 mM tetra-n-butylammonium, pH 5.0: solvent A with 30% methanol. Data are expressed as mean ± SEM of 3–6 independent experiments. ^*#*^*P*< 0.05: significantly different versus vehicle treated control. (*) indicates significant difference versus CIS-treated cells (*P< 0.05). 5-AIQ, 5-aminoisoquinoline; cADPR, cyclic ADP-ribose; CIS, cisplatin; HPLC, high performance liquid chromatography; L-TH, L-thyroxine; NAD^+^, nicotinamide adenine dinucleotide.

It was observed that the pretreatment with L-TH (120nM/4h) significantly decreased the elevated cADPR and the much consumption of CD38 substrate (NAD^+^) as compared with CIS-treated cells; the effect that comparable with the potent PARP inhibitor 5-AIQ pretreatment (Fig 7A and 7B).

### L-TH sensitizes Caco-2 cells to CIS-mediated viability inhibition

**Fig 8:**
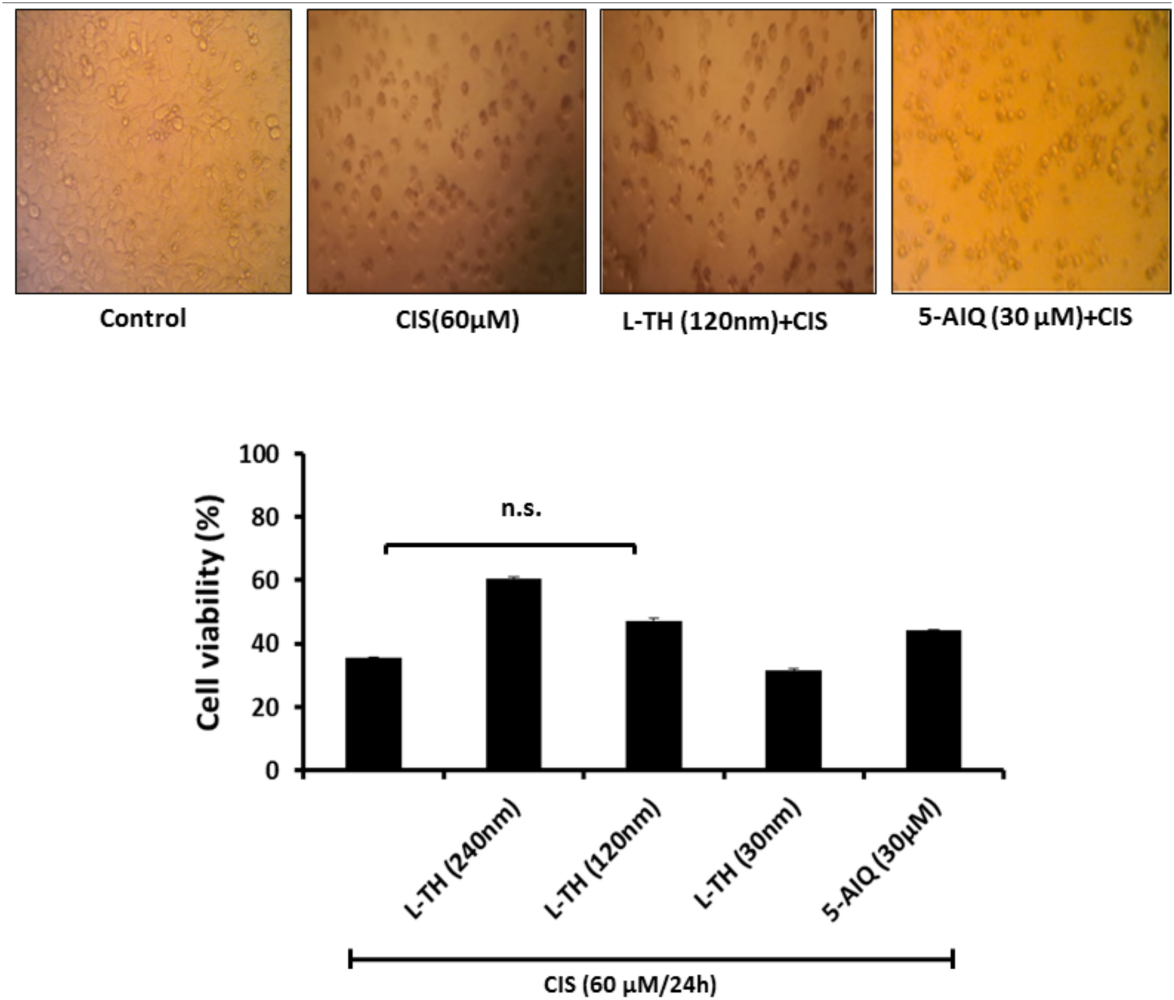
Effect of L-TH on chemosensitivity of cisplatin. Caco-2 cells were pretreated with L-TH (30, 120 and 240 nM) or 5-AIQ (30 μM) four hours before incubation with cisplatin (60μM/24h). Cell viability was determined with MTT assay. 5-AIQ, 5-aminoisoquinoline; CIS, cisplatin; L-TH, L-thyroxine.

The pretreatment with L-TH (120nM/4h) did not interfere the CIS-induced cytotoxic effect as for 5-AIQ. However, L-TH could sensitize such effect in dose decreasing manner (Fig 8).

## Discussion

Nephrotoxicity is a common complication that appeared after treatment of solid tumor with various alkylating agents or abdominal radiotherapy. Many cellular and molecular events are collectively implicated in this phenomenon ranging from oxidative stress, expression of inflammatory biomarkers, PARylation, mitochondrial perturbation, and different forms of cell death (apoptosis, necrosis, or autophagy) that finally cause renal damage (mentioned above). Based on metabolic regulatory effect of thyroid hormones and their receptors wide distribution, we hypothesized that, L-TH (the synthetic analogue of tetraiodothyronine, T4) may control renal injury caused either by repeated administration of CIS or exposure to gamma-radiation. The present work revealed that the pretreatment with L-TH significantly counterbalanced the enhanced levels of renal functions parameters (urea, creatinine and uric acid) for CIS-mediated nephrotoxicity, whereas kidney functions in irradiated mice were slightly improved.

Many essential molecular pathways have been recruited during CIS- and IRR-induced renal tissue damage. One common feature occupied in both insults is the abnormal ROS production. Generally, mitochondria are the benchmark source for ROS production, particularly through respiratory chain complexes activation [26]. The natural defense systems, mainly superoxide dismutases, can decompose the ROS stream and neutralize their peroxides; detoxifies superoxide anion to hydrogen peroxide which scavenged by catalase or glutathione peroxidase at the expense of GSH [27]. These facts were reflected in the current study; whereby the oxidants/antioxidants system was impaired by increased H_2_O_2_ contents with a pronounced low catalase activity in kidneys of CIS-treated or IRR animals. Moreover, oxidative stress has been proved in many studies to be one of the major mechanisms in the pathogenesis of CIS-mediated renal cell damage [1]. Notably, pretreatment with L-TH could neutralize such oxidative imbalance. These observations are in line with the finding of [28]. In general, modified cellular energetics may lead to cell dysfunction or even cell death [26]. Previous studies revealed that IRR exposure or CIS administration radically trigger the consumption of ATP in expense of repairing DNA breaks, the action that finally ends by apoptotic cells [29]. These facts were elucidated in our model and pretreatment with L-TH was a candidate in conserving mitochondrial ATP contents as well as regulating metabolic turnover.

In conjunction with our findings; either CIS or IRR caused cell death; primarily through apoptosis (caspase-3 and Bax expressions) and secondarily by necrosis (LDH release and DNA damage). An increase in both renal necrosis and apoptosis was noticed by the *in vivo* administration of nephrotoxic doses of cisplatin [30] which caused activation of caspase family and the direction of Bax into mitochondria [31]. Furthermore, the renal alteration induced by IRR could be attributed to excessive production of ROS, which will attack membrane lipids and various cellular components including DNA and proteins with a resultant significant cellular damage [32]. In addition to their genomic effects, thyroid hormones have been shown to activate many anti-apoptotic related plasma membrane and cytosol targets initiating non-genomic pathways [33, 34]. L-TH pre-administration effectively inhibited the expression of both Bax and caspase-3, and hence the inhibition of DNA fragmentation. Furthermore, a remarkable decrease in serum LDH was observed in L-TH-pretreated animals as compared with untreated control. In accordance with the present findings, Yang et al. [12] have demonstrated that, the preconditioning with thyroid hormones protected against hepatic damage induced by IRR with an evidence of reducing the pro-apoptotic proteins, Bax and cleaved capase-3, expressions. Another study revealed their capability to stop DNA fragmentation and caspase activation in hepatocytes challenged with Fas/TNFa/ActD [14].

In line with the present findings, variable degrees of necrobiotic changes of the tubular epithelium with marked glomerular histological changes were observed in renal tissue of both models of nephrotoxicity. Similar lesions were more or less observed by previous studies [35, 36]. CIS could enter the renal epithelial cells via certain transports including; OCT2 and to a lesser extent, Ctr1 causing damage to both nuclear and mitochondrial DNA with a resultant production of ROS metabolites. While, renal injury caused by exposure to ionizing radiation is initiated by oxidative injury to deoxyribonucleic acid (DNA) with subsequent alterations in all renal parameters. The pre-administration of L-TH could protect the renal tissue against such harmful effects in both insults.

Accompanying our *in vivo* results, the outcomes extrapolated from MTT Vero cells viability assay revealed a marked time-dependent inhibitory effect of L-TH versus CIS-exerted cytotoxicity. This is comparable with the investigations of **Sukocheva and Carpenter** [14] on mouse model of hepatotoxicity. One previous study had indicated that pretreatment with T3 enhanced CIS-induced nephrotoxicity in rats [37], the finding that was inversely demonstrated in the current work and that might be explained considering time invention. Exposure to gamma radiation was accepted to bring about dose-dependent loss of cell viability [38]. Another study showed that IRR up to 30Gy could stop cell proliferation without changing Vero cell viability for successive fifteen days [39]. High doses of L-TH were found to increase the sensitivity of Vero cells toward gamma radiation; the result that was contradictory with our finding of L-TH on cytotoxicity, hence further investigation on such observation is mandatory in the future.

As mentioned before, one drawback of excessive ROS production is DNA breakdown, the action that causes PARP over activation. This process can deplete cellular NAD/ATP stores and consequently cellular insults may occur [40]; a reason for extreme mitochondrial ATP depletion and cell death promotion (discussed above). This fact was reflected in the current study, whereas CIS resulted in marked depletion of extracted NAD^+^ content and such effect was abolished upon PARP inhibition by a potent PARP inhibitor, 5-AIQ. Recent study has indicated the implication of PARylation in the model of CIS-nephrotoxicity [41]. On other hand, L-TH significantly prevented CIS-caused depletion of NAD^+^ content in Vero cells. This was likely parallel with L-TH ability to decrease the excessive ROS production accompanied by DNA damage, and hence PARP enzyme activation. Another plausible explanation of lower NAD^+^ levels in CIS-mediated renal damage is the up-regulation of ADP-ribosyl cyclase indicated by formation of cADPR in kidney tissue samples and in Vero cells. Data analysis also revealed further mobilization of calcium (high cytosolic calcium levels) upon CIS treatment or IRR exposure as a consequence of cADPR synthesis or extreme ROS formation [42, 43]; the effect that was abolished by pretreatment with L-TH. Despite the anti-apoptotic effect of L-TH on normal Vero cells have been elucidated, the therapeutic potential targeting CIS anti-colorectal carcinoma (Caco-2) has not been disturbed upon L-TH pre-incubation. Our hypothesis is that on the one hand L-TH at dose level 120nM/4h could regulate the mitochondrial biogenesis with apparent protection against cisplatin-induced Vero cells cytotoxicity, while on the other hand it did not affect such toxicity of colorectal carcinoma (Caco-2). In preliminary work, the other dose- and time-dependent protocols of the combined treatment L-TH and cisplatin (e.g. L-TH, 240nM/4h and cisplatin, 60mM/24h) could not pursue the same effect on either two populations; Vero and Caco-2 cells. Thus, although recruitment of L-TH as protector from chemotherapeutic agents’ side effects is a fine candidate, it is not entirely easy to determine a successful treatment regimen. CD38, like PARP, is a bifunctional enzyme that represents a very low Km for NAD+ with a great effect on its metabolism [8]. It was accepted that thyroid hormones were found to stimulate the protective autophagy mechanism via ROS/AMPK/mTOR axis which limits the anabolic pathway and the much ATP consumption [11]. Recent study also proved that CD38/ADP-ribosyl cyclase is an endogenous inhibitor of m-TOR pathway, a promoter of cell proliferation and survival. Furthermore, in oxygen and glucose deprived conditions, cell death will be promoted by stimulation of CD38/cADPR/RyR/Ca2+/CaMK/AMPK [44]. Taken together, the regulatory effect of L-TH on NAD+ /CD38/cADPR/Ca2+ was in line with the plethora of previous investigations that concluded the dual action of thyroid hormones on cell survival and death in different pathological conditions.

In conclusion, the overall data presented here might be relevant in explaining the protective role of L-TH against these *in vivo* and *in vitro* models of nephrotoxicity depending on its master control of mitochondrial ATP, NAD^+^ and ROS reservoirs, which modify the apoptotic cascade through variable interplay between cADP-ribosylation and PARylation.

## Supporting information

S1 Fig IC50 values of CIS-induced cytotoxicity on either Vero or Caco-2 cells as well as dose-dependent effect of L-TH were determined using cell viability (MTT) test. Data are expressed as mean (% of control) ± SEM of six independent experiments.
(TIF)
