# Supplementary figures and images for: L-thyroxine modifies nephrotoxicity by regulating the apoptotic pathway: The possible role of CD38/ADP-ribosyl cyclase-mediated calcium mobilization
Source: PLoS One. 2017 Sep 11;12(9):e0184157. doi: 10.1371/journal.pone.0184157 (PMC5593187; doi:10.1371/journal.pone.0184157)

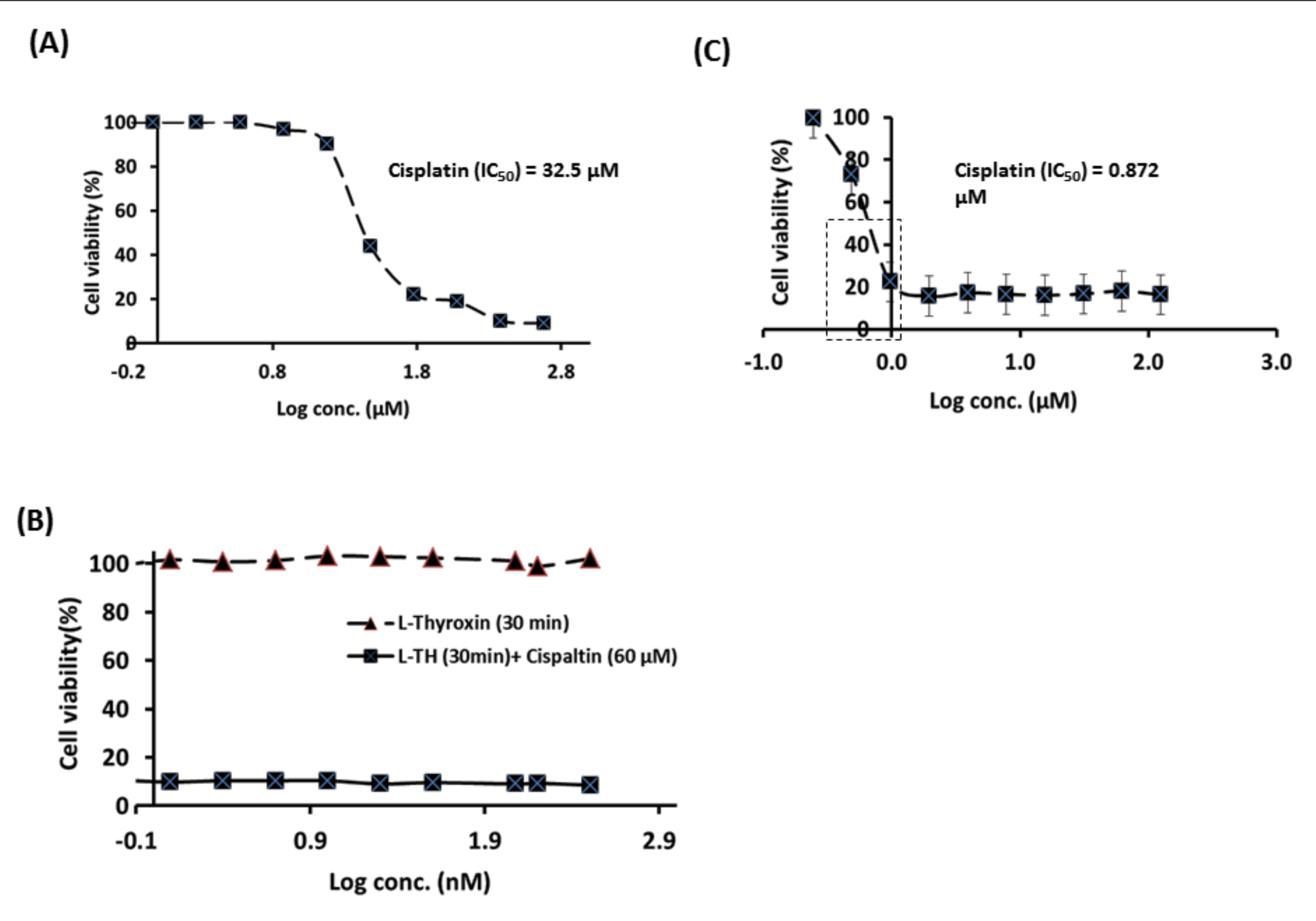

Supplement: S1 Fig — Data are expressed as mean (% of control) ± SEM of six independent experiments. (TIF) [file pone.0184157.s001.tif]
